# Excitatory Post-Synaptic Potential Mimicked in Indium-Zinc-Oxide Synaptic Transistors Gated by Methyl Cellulose Solid Electrolyte

**DOI:** 10.1038/srep38578

**Published:** 2016-12-07

**Authors:** Liqiang Guo, Juan Wen, Jianning Ding, Changjin Wan, Guanggui Cheng

**Affiliations:** 1 Micro/Nano Science & Technology Center, Jiangsu University, Zhenjiang, 212013, China; 2 Jiangsu Collaborative Innovation Center of Photovolatic Science and Engineering, Jiangsu, the Breeding Construction Point of State Key Laboratory of Photovolatic Engineering Science, Changzhou University, Changzhou, 213164, China; 3 Ningbo Institute of Materials Technology & Engineering, Chinese Academy of Sciences, Ningbo, 315201, China

## Abstract

The excitatory postsynaptic potential (EPSP) of biological synapses is mimicked in indium-zinc-oxide synaptic transistors gated by methyl cellulose solid electrolyte. These synaptic transistors show excellent electrical performance at an operating voltage of 0.8 V, *I*_on/off_ ratio of 2.5 × 10^6^, and mobility of 38.4 cm^2^/Vs. After this device is connected to a resistance of 4 MΩ in series, it exhibits excellent characteristics as an inverter. A threshold potential of 0.3 V is achieved by changing the gate pulse amplitude, width, or number, which is analogous to biological EPSP.

For the hardware implementation of neuromorphic computation, various synaptic devices have been proposed to emulate biological synapses, which are the basic units for memory and information processing in neural systems, such as the human brain.^1^,^2^,^3^ Ionic two- terminal devices in which protons (H^+^) provide both memory and output signal have been proposed. These devices exhibited synaptic-like reversible short-term depression, device memory, and can be turned “ON” and “OFF” with as little as 30 nJ of energy per bit.^4^ Much more attention than ever before has been paid to synaptic transistors whose structures are similar to that of the complex dendro-axonic synapse in the neural network.^5^,^6^ For example, an essential synaptic plasticity known as spike-timing-dependent plasticity was realized by programming the timing of a pair of pre- and post-synaptic spikes in MEH-PPV polymer electrolyte-gated synaptic transistors.^7^ Massively parallel signal processing was emulated in carbon nanotube (CNT) synaptic transistors.^8^,^9^,^10^ In these synaptic transistors, ion/electron dynamic interactions observed at the electrolyte/semiconductor interface, are of great significance to the synaptic emulations. Currently, many synaptic functions, such as long-term memory, short-term memory, and excitatory postsynaptic current, are also mimicked by electric-double-layer (EDL) synaptic transistors gated by a proton conducting film.^11^ Broad spectrum proton conducting films, such as nanogranular SiO_2_ films, KH550-GO solid electrolyte, and chitosan membrane, have been proposed as the gate medium of synaptic transistors.^12^,^13^,^14^ Given the significance of gate dielectrics to transistor-based synaptic devices, mobile protons in such proton conductor electrolytes assume an important role in the application of these devices.^15^,^16^ The distribution of mobile protons can be modulated by the voltage spikes applied on the pre-synaptic input terminal. The channel conductance can be tuned by the dynamic interactions between protons and electrons in both electrostatically and electrochemically.

In this study, protonic/electronic hybrid indium-zinc-oxide (IZO) synaptic transistors gated by methyl cellulose (MC) solid electrolyte, as shown in Fig. 1, were demonstrated. This synaptic transistor shows excellent electrical performances. A resistor-loaded inverter was built using this transistor in series with a load resistor, and a voltage gain of 9 was obtained. More importantly, the action potential of EPSP was generated in such inverter by tuning the pattern or timing of the pre-synaptic voltages. Therefore, such transistor-based synaptic devices are of great interest to synaptic electronics and neuromorphic systems.

## Results and Discussion

Figure 2(a) shows a cross-sectional SEM image of the solid MC electrolyte deposited on the n^++^ (100) Si substrate. The solid MC electrolyte thickness was estimated to be ~14.7 μm. An unconsolidated structure was observed and it could provide a larger specific surface area for adsorption of water molecules.^17^ The molecular formula of the solid MC electrolyte is shown in Fig. 2(b). The hydroxyl residues enable the MC electrolyte to absorb water molecules and to achieve high proton conductivity.^18^,^19^ The protons would be from the ionization of water molecules. The mechanism of H^+^ diffusion in the MC solid electrolyte is same as that of other proton conductor films.^20^ The micro pores are supposed to have a columnar structure. Then, each micro pore and solution in micro pore composes a cylindrical solid electrolytic capacitor. When a positive voltage is applied, a large number of protons can move along the direction of the electric field, but protons could be adsorbed by hydroxyl groups on the wall of the micro pores at intervals during proton transport. Figure 2(c) shows the frequency-dependent specific capacitance and the phase angle curves. The specific capacitance increases with decreasing frequency, and it reaches a maximum value (*C*_EDL_) of ~1.7 μF/cm^2^ at 1.0 Hz.^21^ According to the value of the phase angle, the relaxation phenomena can be classified as either capacitive (*θ*(*f*) < −45°) or resistive (*θ*(*f*) > −45°) behavior.^22^ The capacitance–frequency characteristics of the MC electrolyte can be divided into the following three different frequency regions: (1) The capacitive behavior in the high frequency region (*f* > 20 kHz) is attributed to the dipolar relaxation of the solid MC electrolyte. (2) The resistive behavior in the intermediate frequency region (20 Hz < *f* < 20 kHz) originates from dissociated protons migrating away from the MC solid electrolyte in the oscillating electric field. (3) The capacitive behavior in the low frequency region (*f* < 20 Hz) is associated with the formation of the EDL at the interfaces between the MC solid electrolyte and the ITO electrode. Figure 2(d) shows the *Cole*-*Cole* plot of the MC solid electrolyte. The proton conductivity (*σ*) can be calculated from the *Cole*-*Cole* plot with the real axis according to proton conductivity equation:^23^


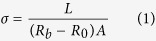


Where *L, A*, and *R*_0_ are the thickness of the MC solid electrolyte, the electrode surface area, and the rig short circuit resistance, which is approximately 30 Ω, respectively. The proton conductivity of the MC solid electrolyte was calculated to be ~1.0 × 10^−3^ S/cm. The high proton conductivity is crucial to the formation of the EDL. The leakage current curve of the solid MC electrolyte is shown in the inset of Fig. 2(d). A voltage (−4 V to 4 V) is applied to the bottom electrode, whereas a voltage of 0 V is applied to the top electrode. The leakage current is below ~2 nA in the voltage range between −4 V and 4 V. Therefore, the performances of the IZO synaptic transistors will not be adversely affected.

Figure 3(a) shows the output characteristics with variation in *V*_gs_ from 0.2 V to 0.8 V in steps of 0.1 V. In the small *V*_ds_ region, the output characteristics curve is in accord with the linear characteristics, thereby indicating Ohmic contact. In contrast, in the high *V*_ds_ region, the channel current of the IZO synaptic transistor reaches the saturation state gradually. The EDL mechanism of the MC solid electrolyte plays an important role in the channel currents of the IZO synaptic transistor. Figure 3(b) shows the corresponding transfer characteristics curve of the device at a fixed *V*_ds_ of 1.5 V. The operating voltage of the synapse is approximately 0.8 V. The energy required to operate the synapse is calculated approximately 16 nJ per bit. The IZO synaptic transistor operates in the n-type depletion mode. An anticlockwise hysteresis loop of ~0.2 V is observed, which was mostly due to the mobile protons in the MC solid electrolyte.^24^ The IZO synaptic transistors exhibit high performances with a large drain current on/off ratio of 2.5 × 10^6^ and a small subthreshold swing of 84.5 mV/dec. A threshold voltage (*V*_th_) of 0.1 V was calculated from the x-axis intercept of the square root of the *I*_ds_–*V*_gs_ plot. The field effect mobility (*μ*) in the saturation region can be estimated to be ~38.4 cm^2^/Vs using the following equation:^25^


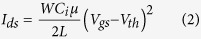


Where *L* (80 μm) and *W* (1000 μm) are the channel length and the channel width, respectively, and *C*_i_ (~1.7 μF/cm^2^ at 1 Hz) is the specific capacitance. Compared with the previous report, the field effect mobility of the IZO synaptic thin-film transistor gated by MC solid electrolyte is higher.^26^,^27^,^28^

A resister-loaded inverter was built by connecting an IZO synaptic transistor with a resistor (4 MΩ) in series, as shown in Fig. 4(a). The source electrode of the IZO synaptic transistor was fixed at 0 V, and the drain electrode was connected to the resistor. A voltage of 1.0 V was applied to the drain electrode. The equivalent circuit is shown in the inset of Fig. 4(b), and the static voltage transfer characteristics (VTC) at the supplied voltage (*V*_DD_) of 1.0 V are shown in Fig. 4(b). When the input voltage (*V*_in_) is lower than −0.25 V, the driver transistor is “OFF.” Therefore, a high output voltage (*V*_out_) of ~1.0 V is obtained. When the input voltage (*V*_in_) is higher than 0.1 V, the driver transistor is “ON,” and therefore, a low *V*_out_ of 0 V is obtained. An abrupt transition in *V*_out_ is observed in the VTC characteristics in response to *V*_in_ of approximately −0.1 V. *V*_out_ could switch within approximately −0.1 V of *V*_in_ variations. The voltage gain (-d*V*_out_/d*V*_in_) of the resister-loaded inverter is calculated to be ~9 from the VTC curve.^29^ It is higher than that (~8.0) of a previously reported electrolyte-gated IZO transistor inverter.^30^ It is noted that because of the proton accumulation, an anticlockwise hysteresis loop of 0.25 V is observed. A series of VTC characteristics are provided in different ranges of input voltage (−0.4 V–0.4 V, −0.6 V–0.6 V, −0.8 V–0.8 V, and −1.0 V–1.0 V), and a series of corresponding anticlockwise hysteresis loops (0.12 V, 0.18 V, 0.24 V, and 0.3 V) are observed as shown in Fig. 4(c). With a larger scanned range, a greater anticlockwise hysteresis loop is observed. Increasingly more mobile protons are induced and accumulated with an increase of the scanned range. The dynamic response of the resister-loaded inverter at a *V*_DD_ of 1.0 V is provided in Fig. 4(d). The device shows good inverter action and response to a low-power square-wave input signal (*V*_in_), switching between −1.0 V and 1.0 V at a frequency of 0.2 Hz. When *V*_in_ is switched between −1.0 V and 1.0 V, a small relaxation time on the order of milliseconds in *V*_out_ is observed. This relaxation behavior is attributed to the migration and accumulation of protons in the MC solid electrolyte, implying that this device can be used for artificial electronic synapses.

EPSP of biological synapses was also mimicked to further confirm the performance of the fabricated device. EPSP is a post-synaptic potential that makes a neuron more likely to fire an action potential. This temporary depolarization of post-synaptic membrane potential, caused by the flow of positively charged neurotransmitters into the postsynaptic cell, is a result of opening ligand-gated neurotransmitter channels.^31^ To simulate this biological phenomenon, the mobile protons, ITO bottom gate electrode, self-assembled IZO channel layer with source/drain electrodes, and conductance of the self-assembled channel layer were regarded as neurotransmitters, pre-synaptic input terminal, post-synaptic output terminal, and synaptic weight, respectively. The resting potential was approximately −70 mV, and the threshold potential was less than 0 mV, as shown in Fig. 5(a). For the resister-loaded inverter based on an IZO synaptic transistor, a threshold potential of 0.3 V was fixed first at a *V*_DD_ of 1.0 V. A series of pulse signals at pulse amplitudes of 0.2 V, 0.6 V, and 1.0 V and a pulse width of 15 ms were imposed on the pre-synaptic input terminal. Because of proton migration and accumulation, the post-synaptic output terminal potential gradually approaches the threshold potential with an increase in the pulse amplitude. It exceeds the threshold potential at the pulse amplitude of 1 V as shown in Fig. 5(b). In addition, at the pulse amplitude of 0.4 V, the post-synaptic output terminal potential gradually approaches the threshold potential and finally exceeds the threshold at a pulse width of 60 ms, as shown in Fig. 5(c). When a series of pulses with a pulse width of 15 ms and pulse amplitudes of 0.4 V, 0.6 V, and 0.8 V were applied, the post-synaptic output terminal potential could exceed the threshold potential with pulse numbers of 16, 3, and 1, respectively, as shown in Fig. 5(d). After a small pulse, a short time is required for the interfacial protons to diffuse back to their equilibrium position. Hence, when other pulses are applied for the pre-synaptic input terminal shortly after the previous pulse, the response of channel current will be enhanced. Thus, mobile protons in the MC solid electrolyte with high proton conductivity and large *C*_EDL_ play a major role in EPSP of EDL synaptic transistors.

## Conclusions

The EPSP of biological synapses was achieved in IZO synaptic transistors gated by MC solid electrolyte. A large EDL capacitance of 1.7 μF/cm^2^ at 1.0 Hz was observed in the electrolyte. The microstructure of the MC solid electrolyte was unconsolidated to the extent that its proton conductivity could reach up to ~1.0 × 10^−3^ S/cm. The operating voltage, the large on/off ratio, and the mobility of self-assembled IZO synaptic transistor were 0.8 V, 2.5 × 10^6^, and 38.4 cm^2^/Vs, respectively. The threshold potential (0.3 V) was achieved by changing the pulse amplitude, width, or number of the pre-synaptic input terminal, which was similar to the EPSP of biological synapses. Therefore, IZO synaptic transistors gated by MC solid electrolyte are promising devices as an alternative artificial synapse.

## Methods

**Figure 1:**
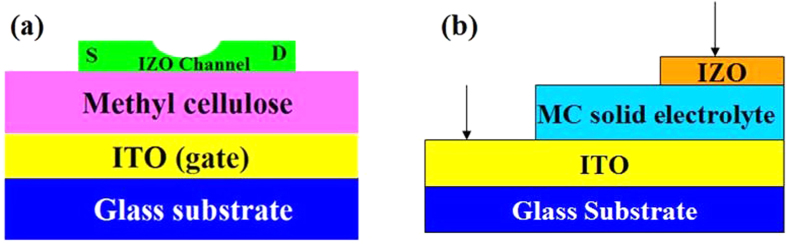
(**a**) Schematic image of an IZO synaptic transistor gated by MC solid electrolyte. The channel length and width are 80 μm and 1000 μm, respectively. (**b**) An ITO/MC/IZO sandwich structure.

**Figure 2:**
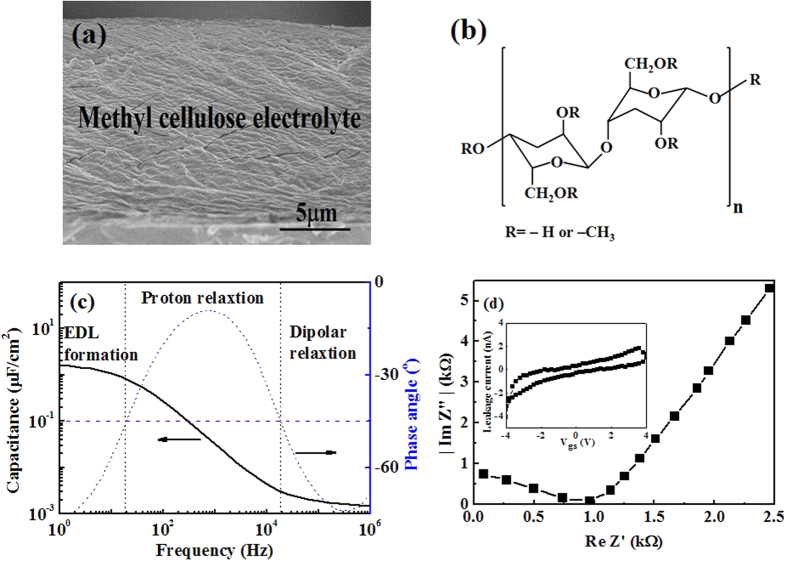
SEM image and electrical characteristic of MC solid electrolyte (**a**) SEM image of MC solid electrolyte on the n^++^ (100) Si substrate. (**b**) Molecular formula of MC solid electrolyte. (**c**) The measured serial capacitance and phase angle versus the frequency of a capacitor based on MC. (**d**) *Cole*-*Cole* plot of MC solid electrolyte tested with an ITO/MC solid electrolyte/IZO sandwich structure. Inset shows an leakage current of MC solid electrolyte. A voltage (−4 V~4 V) is applied on one of metal probes and a voltage of 0 V is applied on the other metal probe.

**Figure 3:**
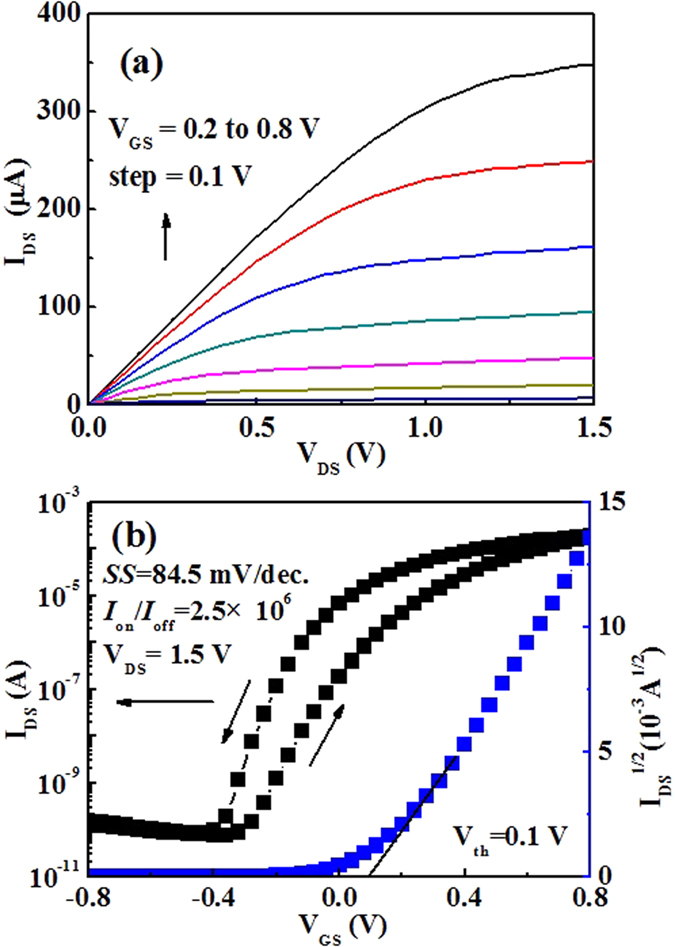
The electrical characteristic of IZO synaptic transistor (**a**) Output characteristic curve of IZO synaptic transistor gated by MC solid electrolyte; The *V*_gs_ step is 0.1 V. (**b**) Transfer characteristic curve of IZO synaptic transistor gated by MC solid electrolyte; the *V*_ds_ is 1.5 V.

**Figure 4:**
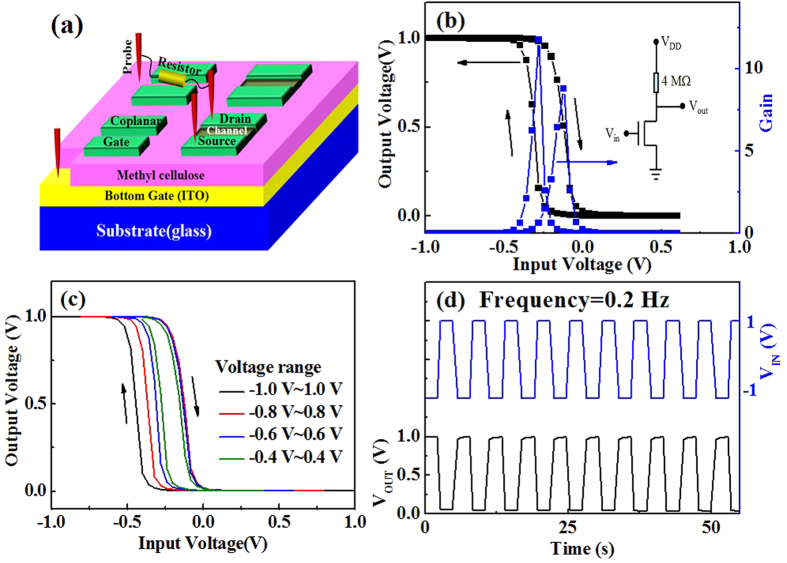
IZO synaptic transistor inverter. (**a**) Test schematic diagram of resister-loaded inverters; the resister-loaded inverter are built by connecting an IZO synaptic transistor with a resistor (4 MΩ) in series. (**b**) Input and output curve of an IZO synaptic transistor inverter; the *V*_dd_ is 1 V. (**c**) Output curve with different scanning ranges of an IZO synaptic transistor inverter; the *V*_dd_ is 1 V. (**d**) Cycle response characteristic curve of an IZO synaptic transistor inverter; the pulse frequency is 0.2 Hz.

**Figure 5:**
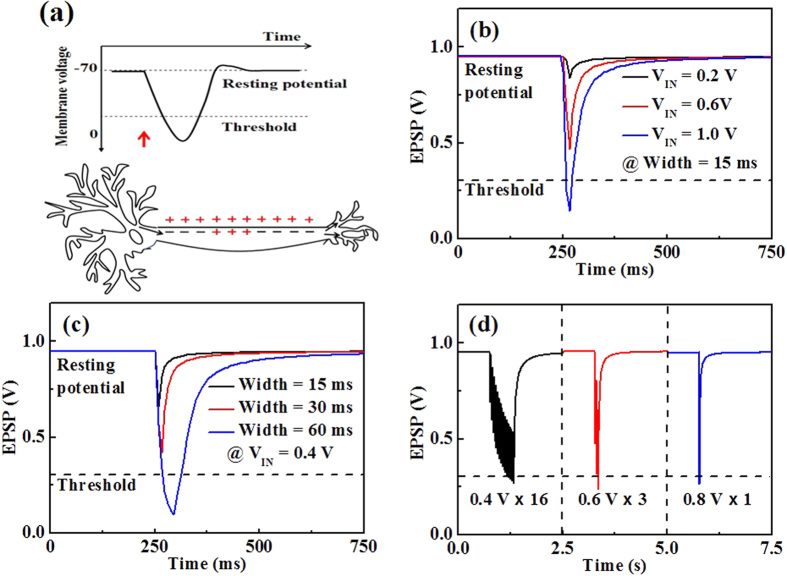
(**a**) The principle of EPSP of biological synapses. (**b**) EPSP with different pulse amplitudes; the pulse width is 15 ms and the *V*_dd_ is set at 1 V. (**c**) EPSP with different pulse widths; the *V*_in_ and the *V*_dd_ is set at 0.4 V and 1 V, respectively. (**d**) EPSP with different pulse numbers. The *V*_in_ are 0.4 V, 0.6 V and 0.8 V, respectively and the *V*_dd_ is set at 1 V.

MC powder (Sinopharm Chemical Reagent Co., Ltd) was mixed into deionized water at 85 °C for 5 min and then cooled in ambient air to obtain a homogeneous solution with a concentration of 1.0 wt%. Then, the MC solution was spin-coated onto an indium-tin-oxide (ITO) glass substrate and dried in ambient air at 50 °C for 2 h. MC solid electrolyte were also prepared on an n^++^ (100) Si substrate to obtain a cross-sectional SEM image by field-emission scanning electron microscopy (FE-SEM) (Hitachi-S4800). 150-nm-thick patterned IZO films were deposited on the MC solid electrolyte by radio-frequency magnetron sputtering. An IZO (In_2_O_3_:ZnO = 90:10 wt%) ceramic was used as the sputtering target. Deposition pressure, working power, and Ar flow rate were 0.5 Pa, 100 W, and 14 sccm, respectively. A thin IZO layer was self-assembled between two patterned IZO films with a distance of ~80 μm due to the diffraction effect.^32^,^33^ Thus, an IZO synaptic thin-film transistor was obtained as shown in Fig. 1(a). The channel length can be defined as the distance between the source electrode and drain electrode. The channel length (*L*) and width (*W*) were 80 μm and 1000 μm, respectively. A thin IZO channel layer can be self-assembled between the source/drain electrodes due to the reflection of the IZO nanoparticles at the mask edge and nanoparticles with a low incident angle lead to extended dimensions. In such processes, the IZO active channel was naturally formed by diffraction during the IZO source/drain electrode deposition without any special process. The thickness of the self-assembled IZO channel depends on the distance between the nickel mask and the substrate. If the mask-to-substrate distance is too small, the IZO channel layer will not be self-assembled. On the other hand, if the mask-to-substrate distance is too large, the self-assembled IZO film is too thick to act as an active channel. A larger negative gate voltage is needed to turn off the transistor with a thicker IZO channel. Generally, the thickness of the IZO channel is estimated to be ~30 nm by using SEM measurement in processes where the mask-to-substrate distance is ~50 μm.

To obtain the capacitance and proton conductivities of the MC solid electrolyte, an ITO/MC solid electrolyte/IZO sandwich structure was also fabricated, as shown as in Fig. 1(b). Both the proton conductivities and the specific capacitance of the MC solid electrolyte were measured by a Solartron 1260 impedance analyzer. All the electrical characteristics of the self-assembled IZO synaptic transistors gated by the MC solid electrolyte were measured by a Keithley 4200SCS semiconductor parameter analyzer. To ensure the experimental accuracy and to reproduce these transistor-based synaptic devices, a large of experiments has been carried out under the same condition, and all the electrical characteristics of the artificial synapse devices were measured in a closed room made by Stalinite. A humidifier and a probe station with a heater were used to maintain the temperature at ~30 °C and humidity at 60%. After all the conditions were s, the testing experiments were carried out repeatedly.

## Additional Information

**How to cite this article**: Guo, L. *et al*. Excitatory Post-Synaptic Potential Mimicked in Indium-Zinc-Oxide Synaptic Transistors Gated by Methyl Cellulose Solid Electrolyte. *Sci. Rep.* **6**, 38578; doi: 10.1038/srep38578 (2016).

**Publisher's note:** Springer Nature remains neutral with regard to jurisdictional claims in published maps and institutional affiliations.
